# 485. Improved Outcome of COVID-19 in CAR T Cell Treated Patients; results of a multicenter study on behalf of the European Society for Blood and Marrow Transplantation (EBMT) Infectious Diseases Working Party and the European Hematology Association (EHA) Lymphoma Group

**DOI:** 10.1093/ofid/ofad500.555

**Published:** 2023-11-27

**Authors:** Per Ljungman, Anne M Spanjaart, Gloria Tridello, Nina Knelange, Rafael de la Camara, Marie Jose Kersten, Stephan Mielke

**Affiliations:** Karolinska University Hospital, Karolinska Institutet, Stockholm, Stockholms Lan, Sweden; Amsterdam University Medical Center, Amsterdam, Noord-Holland, Netherlands; EBMT data office, Leiden, Zuid-Holland, Netherlands; EBMT data office, Leiden, Zuid-Holland, Netherlands; Hospital de la Princesa, Madrid, Madrid, Spain; University of Amsterdam Medical Center, Amsterdam, Noord-Holland, Netherlands; Karolinska University Hospital, Karolinska Institutet, Stockholm, Stockholms Lan, Sweden

## Abstract

**Background:**

COVID-19 has been associated with a very high mortality in patients having received CAR T cell treatment for hematological malignancies. The aim of this study was to investigate whether the outcome has improved over time.

**Methods:**

This is a retrospective analysis of prospective data collected into the EBMT registry regarding risk factors, clinical features, and outcome of patients having received CAR T cell therapy for hematological malignances diagnosed with SARS-CoV-2 infection between March 2020 and December 2022. SARS-CoV-2 was diagnosed by PCR and from January 2021 also antigen tests were accepted. All patients gave informed consent to data collection. The study was approved by the Swedish National Ethical Review Board and by local IRBs as required.

**Results:**

182 patients were included in the analysis; 39 diagnosed in 2020, 35 in 2021, and 108 were diagnosed in 2022. 152 patients were treated for B-cell lymphoma, 19 for acute lymphoblastic leukemia, and 11 for plasma-cell disorders. The median age at COVID-19 diagnosis was 58.7 ys (7.2 - 78.4). The median time from CAR T cell infusion to COVID-19 diagnosis was 7.0 months (1.0 - 42.5). 28% of the patients had lower respiratory tract disease at diagnosis. 27 (25.8%) of patients had received three or more vaccine doses and only 2.2% had received pre-exposure tix/cil. 45.1% required hospitalization, 33.4% required oxygen, and 17% was admitted to ICU. COVID-19 attributable mortality over the entire period was 18.1% with significant improvement over time (43.6% in 2020, 22.9% in 2021%, and 7.4% in 2022).

Survival after COVID-19 diagnosis

**Figure:**
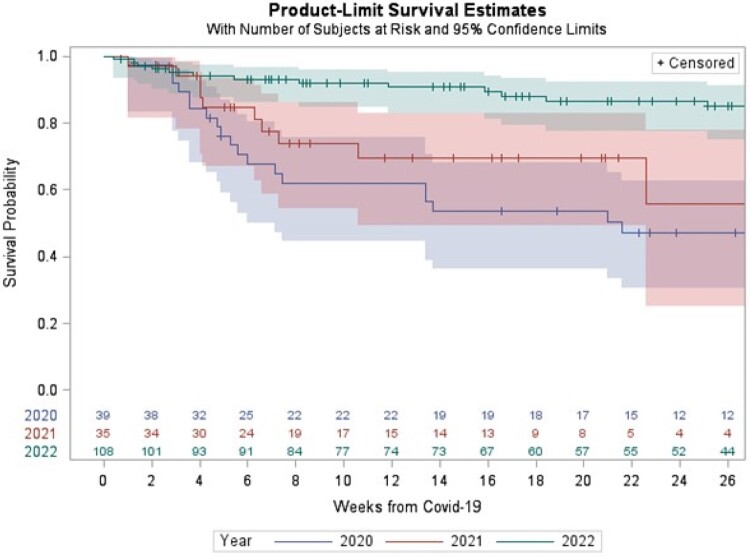

**Conclusion:**

Although the COVID-19 attributable mortality has decreased over time, it remains several times higher than what can be expected in an healthy population of comparable age. Therefore, preventive measures need to be maintained for this vulnerable patient group..

**Disclosures:**

**Per Ljungman, MD; PhD**, AlloVir: Grant/Research Support|Gilead: Honoraria|Moderna: Honoraria|MSD: Honoraria|Takeda: Honoraria **Rafael de la Camara, MD**, AstraZeneca: Advisor/Consultant|Moderna: Advisor/Consultant|MSD: Advisor/Consultant **Marie Jose Kersten, MD, PhD**, BMS: Advisor/Consultant|BMS: Honoraria|Celgene: Advisor/Consultant|Celgene: Grant/Research Support|Celgene: Honoraria|Kite/Gilead: Advisor/Consultant|Kite/Gilead: Grant/Research Support|Kite/Gilead: Honoraria|Kite/Gilead: Travel|Miltenyi Biotech: Advisor/Consultant|Miltenyi Biotech: Honoraria|Miltenyi Biotech: Travel|Novartis: Advisor/Consultant|Novartis: Honoraria|Novartis: Travel|Roche: Advisor/Consultant|Roche: Grant/Research Support|Roche: Honoraria|Takeda: Grant/Research Support **Stephan Mielke, MD, PhD**, BMS: Honoraria|Celgene: Honoraria|Janssen: Honoraria|Kite/Gilead: Honoraria|Mendes: Honoraria|Miltenyi: Honoraria|Pfizer: Honoraria

